# Bridging the Gap: First Clinical Experience With Linear Accelerator (LINAC) Radiosurgery Using Automated HyperARC for Brain Metastasis in a Low-Resource Setting in Pakistan

**DOI:** 10.7759/cureus.99622

**Published:** 2025-12-19

**Authors:** Aqueel Shahid, Tabinda Sadaf, Asma Rashid, Raheel Mukhtar, Zeenat Sattar, Sadia Anjum, Muhammad Anas Tahseen Asar, Umair Zafar, Haniya Rizwan

**Affiliations:** 1 Radiation Oncology, McGill University, Montreal, CAN; 2 Clinical and Radiation Oncology, Shaukat Khanum Memorial Cancer Hospital and Research Centre, Lahore, PAK; 3 Medical Physics, Shaukat Khanum Memorial Cancer Hospital and Research Centre, Lahore, PAK; 4 Medical Physics, Saleem Memorial Hospital, Lahore, PAK; 5 Science, Lahore Grammar School, Lahore, PAK

**Keywords:** brain metastases, ha-vmat, linac, low- and middle-income countries, radiation oncology, stereotactic radiosurgery

## Abstract

Introduction

Brain metastasis is becoming increasingly common in cancer patients. Over the past decade, stereotactic radiosurgery (SRS) and fractionated stereotactic radiotherapy (fSRT) have become the cornerstone of treatment for limited brain metastases. Sophisticated linear accelerator (LINAC)-based HyperArc volumetric modulated arc therapy (HA-VMAT) systems offer the unique advantage of high-precision radiotherapy. The purpose of this study is to present our first clinical experience with the HA-VMAT planning approach for brain metastases treated at our hospital.

Methods

This retrospective study included 72 patients with 1-5 brain metastases treated at our institution with SRS/SRT using HA-VMAT. A dosimetric evaluation of clinical characteristics and outcomes was carried out.

Results

From April 2020 until June 2021, a total of 86 brain metastases in 72 patients were treated. The median age was 45 (range 18-75). The most common histology was breast cancer (n = 48 (66.7%)). Sixty-two (86.1%) patients had a single brain metastasis. Thirty-two (44%) patients were treated with single-fraction SRS, while 40 (65%) received fSRT. The radiobiological equivalent dose in 2 Gy fractions (EQD2) ranged from 50.4 to 81.6 Gy for SRS and from 37.25 to 60.0 Gy for fSRT. The median GTV and PTV volumes for a single fraction were 1.98 cc (range 0.13-13.3) and 4.49 cc (range 0.64-21.20), respectively. The mean PTV was 10.96 cc (range 2.25-34) and 19.04 cc (4.40-75.88). Complete response was observed in 12.5% of the patients. The median follow-up was 17 months (range 1-38 months). The progression-free survival at 1 year was 54.6%. Only one patient developed radiation necrosis.

Conclusion

Brain metastases can be safely and effectively treated with SRS/fSRT using HA-VMAT, which decreases the likelihood of local complications and improves local control.

## Introduction

Brain metastases develop in approximately 20-40% of patients with cancer over the course of their illness [1]. A range of treatment options is available, including surgical resection, whole-brain radiotherapy (WBRT), stereotactic radiosurgery (SRS), corticosteroids, and supportive care. The choice of therapy is influenced by several patient-specific factors, such as the number, size, and location of lesions, the patient's performance status, and the burden of systemic disease. Increasingly, the potential neurocognitive side effects of WBRT, such as memory impairment and reduced executive function, have led clinicians to favor focal treatments, such as SRS, especially in patients with a limited number of brain metastases and good functional status [2,3]. These considerations underscore the importance of individualized treatment planning that balances local control with the preservation of quality of life.

Over the past decade, SRS or fractionated stereotactic radiotherapy (fSRT) has become the cornerstone of treatment for limited brain metastases [4]. SRS offers several benefits over WBRT, including a high probability of disease control and limiting radiation to the normal brain, which helps minimize neurotoxicity. The sharp decrease in dosage from the tumor surface is crucial in stereotactic radiosurgery. The guidelines for the management of brain metastasis published by the American Society for Radiation Oncology consider SRS as the primary treatment to improve survival and quality of life in patients with Brain metastasis [5].

Several techniques are available to deliver this, including Gamma Knife, Cyber Knife, and linear accelerator (LINAC) based systems. Sophisticated LINAC-based systems offer the unique advantage of high-precision radiotherapy with accuracy [6,7]. Recent technical advances in LINACs, including frameless immobilization, high-definition multi-leaf collimators (MLCs), Robotic Couch, and flattening filter-free mode, are significantly more efficient in offering shorter treatment times, better dose conformity, and gradient in comparison to conventional LINACs [8-10].

Recently, Varian Medical Systems (Palo Alto, CA, US) offered a new advanced solution to fulfill the demands of dose delivery for SRS HyperArc volumetric modulated arc therapy (HA-VMAT), which includes automated settings for isocenter and beam arrangement, including no coplanar and appropriate collimator angles according to tumor location. This helps deliver a conformal dose to the target while achieving a sharp dose gradient in the surrounding normal structures [11]. Nevertheless, only a limited number of studies have reported the dosimetric benefits and clinical implementation of HA-VMAT in middle- and low-income countries.

In resource-constrained countries, such as Pakistan, the adoption of advanced radiotherapy techniques remains significantly limited because of several critical barriers. These include a lack of modern linear accelerators capable of delivering sophisticated techniques such as HyperArc VMAT, insufficient numbers of trained radiation oncologists, physicists, and dosimetrists, and a generally high patient-to-machine ratio, which overwhelms the available infrastructure [12,13]. Additionally, the upfront and maintenance costs associated with HyperArc-capable machines and treatment planning systems are prohibitively high for many public-sector institutions.

HyperArc VMAT, a high-precision stereotactic technique primarily used for intracranial tumors, requires not only state-of-the-art hardware but also advanced immobilization devices, image-guided radiotherapy (IGRT) capabilities, and rigorous quality assurance protocols--all of which are either scarce or inconsistently available in many treatment centers across Pakistan [14]. The lack of dedicated training programs and limited exposure to modern contouring, planning, and verification workflows further delay the integration of such techniques into routine practice. Consequently, patients who could benefit from the dosimetric advantages and improved outcomes of HyperArc VMAT often receive suboptimal or less conformal treatments. This perpetuates the disparity in access to precision oncology between high-income and low- and middle-income countries (LMICs), undermining global efforts to ensure equitable cancer care [15].

Although the Gamma Knife is widely regarded as the benchmark for intracranial SRS, its high cost and specialized infrastructure limit its feasibility in LMICs. In contrast, LINAC-based SRS offers a more adaptable and cost-effective alternative, particularly in resource-constrained settings. At our institute, one of the most modernized cancer centers in the country, equipped with linear accelerators and advanced radiation technologies, including image-guided radiotherapy (IGRT), the implementation of LINAC-based SRS was both feasible and efficient. By leveraging existing infrastructure and a trained workforce, we integrated SRS into our routine clinical workflow without major operational disruptions.

For radiation therapy technologists (RTTs), familiarity with LINAC systems minimizes the need for extensive retraining, further streamlining the adoption process. This approach not only enhanced our institutional capacity for delivering high-precision treatments but also helped bridge the significant gaps in access to stereotactic services that persist across the country. By optimizing available resources and modern technology, the LINAC-based SRS at our center has improved treatment equity and set a replicable model for other institutions striving to expand access to precision radiotherapy in similar LMIC contexts.

This study aimed to report our initial experience of clinical and dosimetric parameters for target and normal tissue for the HA-VMAT planning approach in brain metastasis treated at our institution.

## Materials and methods

After receiving approval from the Institutional Ethics Committee of Shaukat Khanum Memorial Cancer Hospital and Research Centre, Lahore, eligible patients with confirmed systemic malignancies, excluding small cell carcinoma and lymphoma histologies, were analyzed. All patients were aged ≥ 18 years and had no prior cranial radiation. The inclusion criteria required a contrast-enhanced MRI demonstrating one to five brain metastases, with the largest lesion measuring up to 4 cm in diameter and additional lesions not exceeding 3 cm, with no evidence of meningeal disease or hydrocephalus. Each case was reviewed in conjunction with neurosurgery to assess resectability. Lesions located in eloquent brain regions or the deep cortex were deemed unresectable. Patients with an Eastern Cooperative Oncology Group (ECOG) score of 3 and beyond [16], uncontrolled widespread systemic disease, or a life expectancy < 6 months were excluded. Each patient underwent a planning computed tomography (CT) scan--a helical scan with 1 mm slice thickness and spacing using the Encompass™ SRS Fibreplast® System with IntegraBite (CQ Medical, Avondale, PA, US). In addition, all patients underwent a 1.5-3 Tesla MRI of the brain. The imaging protocol included 1 mm slice thickness T1-weighted imaging with and without gadolinium (voxel size 1.1 × 1.1 × 1.3 mm^3^) and T2-weighted imaging. The time between the CT and MRI scans was less than 72 h. The CT and MRI datasets were registered using deformable registration with a focus on the target area.

Two certified radiation oncologists independently defined the gross target volumes (GTVs) as the region of contrast enhancement on T1-weighted MRI, and an isotropic 2 mm margin was added for individual planning target volume (PTV). A consensus for the volume was achieved in the SRS planning tumor board, and this was approved for planning.

The dose and fractionation were determined based on the volume of the largest GTV and normal brain (excluding GTV volume) dose constraints and proximity to nearby organs at risk (OAR), especially the brainstem and optic pathway. A 2 mm planning organ-at-risk volume (PRV) was used for OARs, especially the optic chiasma, brain stem, and spinal canal. OAR constraints were based on dose limits for these organs in quantitative analyses of normal tissue in the clinic (QUANTEC) and the hypofractionation table on the wall [17,18].

Treatment planning was performed using the Eclipse planning system (Varian Medical Systems), with dedicated HyperArc algorithms. The planner selects from a predefined set of optimized beam geometries, often with the automated avoidance of OARs such as the brainstem, optic nerves, and cochlea. The prescription isodose was selected between 75% and 85% based on the proximity to the OARs and the required dose fall-off. The dose rate in all patients was 1400Mu/min. The gantry speed and multileaf collimator (MLC) positions were dynamically modified during the arc rotations using VMAT. Collision checks and patient-specific quality assurance (QA) were performed before treatment delivery. To assess the quality of the plan, the following dose conformity parameters were computed: 1. Radiation Therapy Oncology Group (RTOG) Conformity Index (CI): It describes how closely the area receiving a high dose matches the intended treatment volume, typically the PTV; 2. Paddick Conformity Index (CI): The Paddick conformity index was proposed in 2000 by Paddick and aims to provide an objective method of plan quality and eliminate false scores; 3. NEW Conformity Index (NCI): The Paddick conformity index was altered to its reciprocal, allowing it to align with the ratio of Prescription Isodose Volume (PI) to the Target Volume TV, which is termed as (PITV) (when the PITV is greater than 1, which is nearly always the case); 4. Paddick Gradient Index (GI): For radiosurgery in the brain, extra parameters may be considered, such as the dose gradient index GI [19].

All patients were followed up six weeks post-radiation for clinical evaluation. The patient underwent the first MRI 12 weeks post-radiotherapy, followed by quarterly MRI and clinical follow-up for the first year and biannual follow-up thereafter.

## Results

From April 2020 to June 2021, a total of 86 brain metastases in 72 patients treated with HA-VMAT SRS and radiotherapy were evaluated retrospectively. The patient and disease characteristics are summarized in Table 1. The median age of the patients was 45 (18-75), with the majority (56; 77.8%) being female. Forty-eight (66.7%) patients were classified as ECOG-1, and 56 (77.7%) were not on steroids at presentation. The most common histology was breast cancer (48; 66.7%), followed by lung cancer (8; 11.1%). Four patients underwent surgery followed by a cavity stereotactic boost.

**Table 1:** Patient characteristics ECOG = Eastern Cooperative Oncology Group, RPA = recursive partitioning analysis

Gender	
Male	16 (22.2%)
Female	56 (77.8%)
ECOG [16]	
0-1	48 (66.7%)
>2	24 (33.3%)
RPA	
1	37 (51.4%)
2	35 (48.6%)
Presentation	
Metachronous	71 (98.6%)
Synchronous	1 (1.4%)
Primary Disease	
Breast	48 (66.7%)
Lung	8 (11.1%)
Others	16 (22.2%)
Surgical Resection	
Yes	6 (8.3%)
No	66 (91.7%)
Location	
Supra-tentorial	47 (65.3%)
Infra-tentorial	21 (29.2%)
Brain stem	4 (5.55%)
Number of Lesions	
1	62 (86.1%)
2-4	8 (11%)
5	2 (2.7%)
Number of Fractions	
1	32 (44.4%)
03-May	40 (55.6%)

Of the 72 patients, 62 (86.1%) had a single brain metastasis. A total of 32 (44%) patients were treated with single-fraction SRS, while 40 (65%) received fSRT either due to a close proximity to critical structures or due to an inability to achieve V12 for normal brain tissue. The radiobiological equivalent dose of 2 Gy (EQD2) using α/β ratios of 10 for SRS and SRT was in the ranges of 50.4-81.6 and 37.3-60.0, respectively. For a single fraction, the mean prescribed dose was 21 Gy (range 18-24 Gy), and for SRT, it was 25 Gy prescribed at a median isodose of 80% (range 69.5-85).

**Figure 1:**
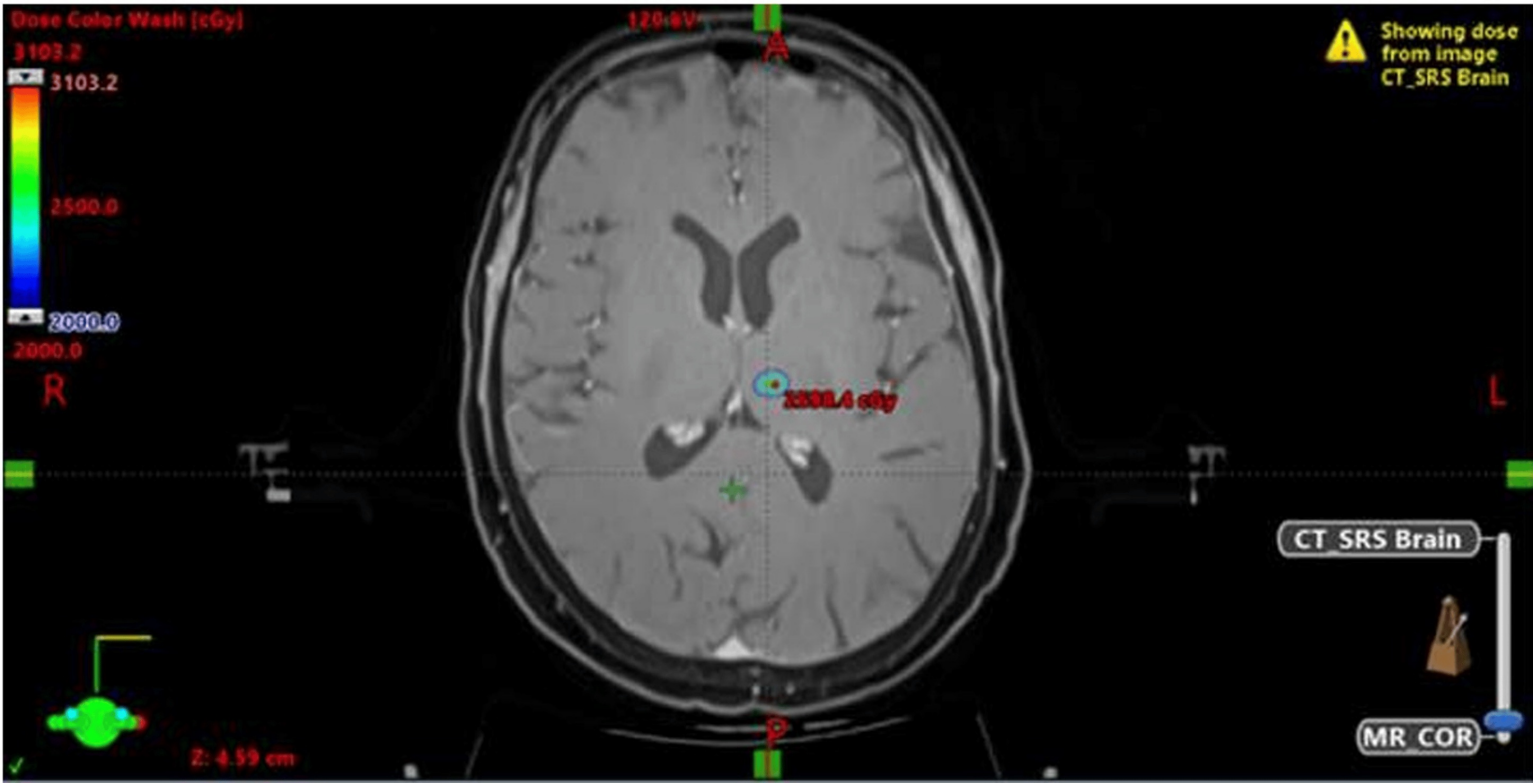
Dose distribution in a patient treated for a single brain metastasis in the deep lobe

Table 2 summarizes the dosimetry parameters of the HyperArc treatment plan. The median GTV and PTV volumes in a single fraction were 1.98 cc (range 0.1-13.3) and 4.49 cc (range 0.7-21.2), respectively, while in fSRT, they were 10.96 cc (range 2.3-34) and 19.04 cc (range 4.4-75.9). The median GTV coverage, defined as the target dose covering the tumor volume, was 104% (range 90.0-125.9) while the mean PTV coverage was 92% (87.2-113.3). The median PTV D2cc and PTV D98cc coverage were 121.3% (range 98.4-157.6) and 101.9% (range: 77.1 - 111.8), respectively. The median coverage of the PTV D50 cc was 111.17% (94.5-124.2) (Figure 1 shows the dose distribution).

**Table 2:** Summary of organ-at-risk sparing and indices used in plan evaluation in multiple fractions RTOG = Radiation Therapy Oncology Group, GTV = gross tumor volume, PTV = planning target volume

Parameter	Median	Range
Isodose Line	80	69.5-90
GTV Coverage (%)	103.97	90.02-125.93
PTV Coverage (%)	94.75	87.23-113.3
RTOG Index [19]	1.03	0.54-2.06
PADDICK Index [19]	0.93	0.30–1.66
Gradient Index [19]	2.8	2.10–5.75
Conformity Index [19]	1.06	0.60–3.33
Total Beam on Time (Sec)	202	146–556
Total Monitor Units (MU)	4025	897–12921
Multiple Fractions Dose (Gy)	27.93	21–35
Multiple Fractions GTV Volume (cc)	10.96	2.52–34.00
Multiple Fractions PTV Volume (cc)	19.04	4.40–75.88
Normal Brain V15 Gy (%)	26.7	6.98–46.50
Normal Brain V18 Gy (%)	20.5	4.81–32.10
Single Fraction Dose (Gy)	21.03	18–24
Single Fraction GTV Volume (cc)	1.98	0.13–13.30
Single Fraction PTV Volume (cc)	4.49	0.64–21.20
Normal Brain V12 Gy (%)	7.88	1.63–10.0

The Conformity scores were also within international standards. The median Conformity Index (CI), RTOG CI score, PADDICK score, and Gradient Index (GI) were 1.06 (range 0.60-3.33), 1.03 (0.54-2.06), 0.93 (range 0.30-1.66), and 2.80 (range 2.10-5.75), respectively. The median monitor units (MUs) consumed were 4025 (range 897-12921), and the beam on time was 4.3 min (range 2.4-9.2 mins) per treatment, with the overall estimated dose-to-door treatment time being less than 15 min.

The patients were positioned by applying 6DoF couch corrections after performing cone beam computed tomography (CBCT) from the original isocenter before treatment delivery. The mean relative rotational setup errors for pitch, yaw, and roll were -0.16° (range -2.2-1.3), 0.02° (range -1.5-1.9), and 0.2° (range -1.9-2.4), respectively.

**Figure 2:**
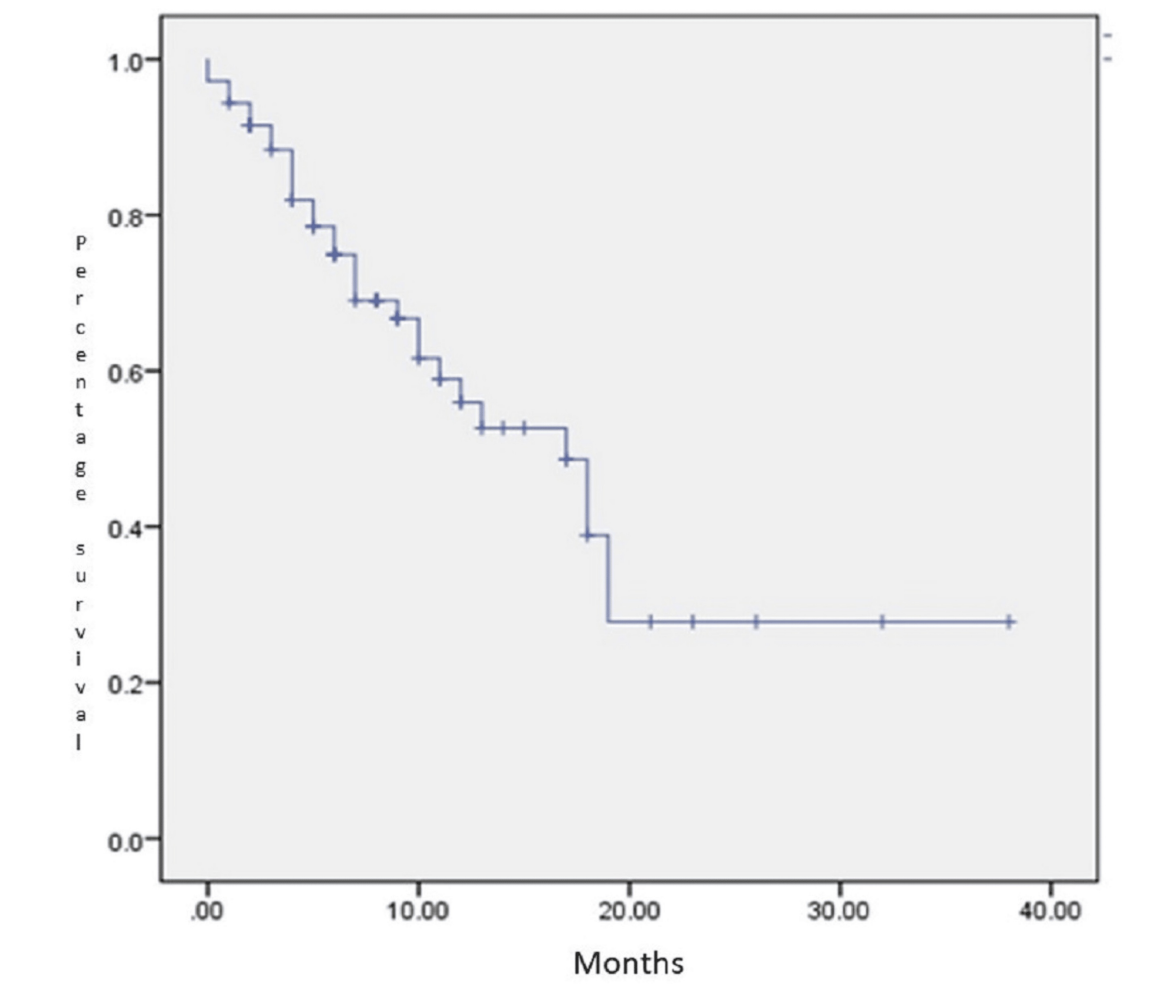
Overall survival

The median follow-up in our study was 17 months (1-38 months). The progression-free survival at 1 year was 54.6%, including intracranial and extracranial disease. Complete response was seen in 12.5%, stable disease in 23.6%, and progression in 12.5% at the first response assessment scan based on the RECIST criteria (response evaluation criteria in solid tumors). Local control was achieved in 65 (90.3%) patients, with only 7 patients developing progression at the radiation site. Most of the patients had distant metastasis (Table 3). Median time to progression was 9.5 months. At the time of analysis, 48 out of 72 (66.67%) patients were alive. Overall survival was 78% at 1 year and 58.3% at 2 years (Figure 2).

**Table 3:** Site of progression

Site	N	Percent
Extracranial	5	16.1%
Intracranial	12	38.7%
Intralesional	7	22.6%
Extra and intracranial	7	22.6%

There were no major acute or late toxicities at the time of analysis; only one patient developed symptoms and radiological evidence of radio necrosis requiring intervention.

## Discussion

SRS is the preferred treatment for individuals with circumscribed brain metastases and reduces the risk of neurocognitive impairment compared to whole brain irradiation [2,20]. While the efficacy of LINAC-based frameless SRS using HA-VMAT is well-documented for extracranial sites, such as the lung, liver, and spine, its application for intracranial lesions, particularly in LMICs, remains limited due to infrastructure, training, and cost-related barriers. However, delivering advanced care is only one aspect of comprehensive cancer management. Follow-up and post-treatment surveillance in LMICs present ongoing challenges. Many patients are lost to follow-up due to socioeconomic barriers, geographical limitations, lack of transportation, or poor health literacy, which makes it difficult to conduct research in these areas and thus limits the data that is available. This paper presents the inaugural clinical experience of employing SRS/SRT for brain metastases with the novel HyperArc non-coplanar mono-isocenter approach in Southeast Asia.

The median follow-up duration of our study was 17 months. In our series, the 1-year local control rate for lesions less than 2 cm in equivalent sphere diameter is 65%, compared to 47% for lesions greater than 2 cm, which aligns closely with existing research [21]. Retrospective analyses of SRS for minor brain metastases indicate 1-year local control rates between 86% (20-24 Gy SRS for metastases ≤ 1 cm in diameter) to 56% (18 Gy SRS for brain metastases with a median diameter of 1.0 cm) [22]. Chang et al. documented 135 patients with brain metastases (BM) measuring less than 5 cc in volume (about 2 cm in diameter), where all 153 assessable BM were subjected to a prescribed dose (PD) of 20 Gy or above. The actuarial local control rates at one and two years were 86% and 78%, respectively, for lesions ≤1 cm, in contrast to 56% and 24%, respectively, for lesions beyond 1 cm (P = 0.0016) [23].

In our institution, the median progression-free survival was 62.7% in the SRS group and 45.9% in the fSRT group. Since it was a risk-based decision to treat small metastases without proximity to critical organs at risk with SRS and larger ones with fSRT, a direct comparison of the efficacy is not feasible. The systematic review conducted by Wiggenraad et al. showed that, irrespective of the dose prescribed, the 6-month overall local control is 80%. While the local control at 20 months was 80%, 60% and 50 % for doses >21 Gy, >18 Gy, and <15 Gy, respectively [24].

In our study, distant brain failure at 56.4% at 1 year when single-fraction and fractionated SRS were analyzed together. This high failure rate is mainly due to the lack of availability of novel agents post-SRS treatment due to financial and regulatory limitations, which have a significant role in establishing long-term disease control.

It is established that whole brain irradiation causes neurocognitive decline that may affect patients’ quality of life [25]. An earlier study demonstrated that the mono-isocenter technique notably enhanced the gradient index (GI) and CI in comparison to a multi-isocenter HA-VMAT plan. Additionally, the improvement in gradient index translated to a notable decrease in doses to normal brain measured by (brain V12), particularly for doses in the range of 2-5 Gy [26]. The enhanced brain sparing attainable with HA-VMAT translates into better neurocognitive functions in patients.

Although the follow-up period was relatively brief, the occurrence of radiation necrosis, which is a dose-limiting toxicity in this series, was low, with just 1 case reported out of 72 treated patients. This singular instance was much less than what has been documented in other comparable series [27] and may be attributed to the sharper GI offered by HyperArc™ or the implementation of strict limits for normal brain tissue, as noted in previous studies [28]. Though this rather unexpected outcome could also be attributed to the lack of long-term follow-up and missed interpretations on imaging, which requires dedicated MRI sequences and expert reporting.

Our findings contribute to the growing body of evidence supporting LINAC-based SRS as a viable and effective treatment for brain metastases in resource-limited environments. Widespread adoption of such techniques, combined with policy-level support for infrastructure development and patient retention strategies, may help reduce disparities in neuro-oncology care across LMICs. This method also serves as a quick and efficient way for planning and administering these highly conformal, complex, advanced local-ablative brain therapies.

## Conclusions

SRS-fSRT combined with HA-VMAT is a safe and effective treatment option for patients with brain metastases, offering improved local control that may, in selected cases, translate into prolonged survival and reduced neurocognitive decline. Our experience demonstrates that implementing stereotactic radiosurgery using HyperArc technology in an LMIC is both feasible and clinically impactful. Despite resource constraints, treatment quality, dosimetric accuracy, and clinical outcomes were comparable to those reported from high-income centers, highlighting the potential for advanced radiotherapy techniques to be successfully adopted in resource-limited settings.
